# The Role of Program Implementation Quality in Family-Focused Substance-Use Prevention for Youth with Multiple Risks: PROSPER Project

**DOI:** 10.1007/s11121-026-01929-9

**Published:** 2026-05-23

**Authors:** Yoon S. Hur, Sarah M. Chilenski, Patrick R. O’Neill, Damon E. Jones, Richard L. Spoth, Mark E. Feinberg, D. Max Crowley

**Affiliations:** 1 Edna Bennett Pierce Prevention Research Center, Pennsylvania State University, University Park, PA USA; 2 Iowa State University, Ames, IA USA

**Keywords:** Implementation quality, Fidelity, Adherence, Facilitation quality, Participant responsiveness, Substance-use prevention, Youth with risks

## Abstract

Programming to prevent behavioral problems can effectively build youth skills and enhance bonding to parents and prosocial peers to delay substance use for adolescents, an important challenge for adolescents given the links between early adolescent experimentation and the chance of developing problems related to substance use as an adult. For youth at higher risk, the effective delivery of such programs could be particularly critical. This study examined the impact of implementation outcomes of family-focused evidence-based prevention programming in sixth grade on substance use in ninth grade based on youths’ initial levels of risk. Three implementation outcomes were assessed: (1) adherence, (2) facilitation quality, and (3) participant responsiveness. Using multi-level analysis of data from two cohorts across 14 school districts, we examined whether community-level implementation quality moderated the relationship between individual-level baseline risk and later substance use. Results showed higher implementation quality, particularly facilitation quality and participant responsiveness, was significantly associated with lower substance use among high-risk youth. Notably, high implementation quality was significantly associated with lower substance initiation index scores for high-risk students (*p* < 0.05). Similar results were also found for the initiation of marijuana and cigarette use, as well as for alcohol consumption in the past month.

**Supplementary information:**

The online version contains supplementary material available at 10.1007/s11121-026-01929-9.

## Introduction

A strong body of literature demonstrates the importance of both peers and parents in regard to adolescent decisions to use a variety of substances (Allen et al., 2003). Given the importance of bonding with both parents and positive peers, school-based and family-focused programs have been developed to enhance their protective influences during adolescence (Bahr et al., 2005; Steinberg et al., 1994). Programs that combine both parents and peers, such as the Iowa Strengthening Families Program:10–14, may be especially powerful influences on adolescent behavior (Proctor et al., 2011). It is at this confluence that prevention scientists and practitioners came together to create PROSPER (PROmoting School-community-university Partnerships to Enhance Resilience; Spoth et al., 2004), a community-level delivery system for school-based and family-focused evidence-based substance misuse prevention programs.

It is widely recognized that implementation quality is necessary for replicating positive participant outcomes observed in evidence-based prevention programs (Glasgow et al., 2019a, b; Proctor et al., 2011). Additionally, the characteristics of the intervention recipients have been identified as important when determining participant outcomes (Damschroder et al., 2022). Building on this literature, this study takes a deeper look at PROSPER program outcomes by testing heterogeneity in program effects. We examine the complex interplay among implementation outcomes related to the family-focused program (i.e., Iowa Strengthening Families Program: 10–14), characteristics of intervention recipients, and youth substance use outcomes using the PROSPER intervention trial data (Spoth et al., 2011a, b). The aims are twofold. First, we investigate how initial youth risk level and three measures of fidelity, including adherence, quality of program delivery, and participant responsiveness, are linked to adolescent substance use outcomes. Second, we examine how levels of initial youth risk and implementation outcomes come together to impact youth behaviors to ask, how do participant outcomes vary for higher- and lower-risk youth who experience different levels of program quality?

### Intervention Recipient Characteristics

In a universal program, such as the programs embedded with the PROSPER design (Spoth et al., 2004), all youth within a particular setting become program participants (Offord, 2000), or at least are invited to participate. No one, regardless of preexisting characteristics, is singled out for participation or told they cannot participate (Offord, 2000). This level of inclusiveness has many advantages such as improving risk/protection for an entire community and avoiding stigma from participation (Offord, 2000), and several studies summarized by Greenberg and Abenavoli (2017) have demonstrated effects for youth across the risk spectrum. However, some scholars continue to describe potential disadvantages as well. They argue that universal programs may not be sufficiently strong to influence high-risk individuals, though another concern is that universal programs can do little to support positive outcomes for low-risk individuals (Offord, 2000; Spoth et al., 2006). Even more so, one worry of universal, voluntary, family-focused programs, such as the Iowa Strengthening Families Program:10–14 that was part of the PROSPER trial, is that the families most in need of the program will not attend, and some evidence has supported this concern as valid (LoBraico et al., 2021; Rosenman et al., 2012; Spoth et al., 2000).

These are important issues to tease out when implementing evidence-based programs, as these characteristics, either separately or together, can lead to variability in the youth program experience (Montero-Marin et al., 2023) and in youth program outcomes (Gottfredson et al., 2015; Spoth et al., 2007, 2013). Prior research has identified early risk indicators of developing later substance misuse, such as early experimentation with substance use (Arthur et al., 2007; Ellickson et al., 2003; Grant & Dawson, 1997; King & Chassin, 2007), coming from a single-parent-headed household (Amato, 2001; Flewelling & Bauman, 1990; Hoffmann, 2002), and poverty (Arthur et al., 2007). In addition, another study found that the occurrence of multiple risk factors in a youth’s life can moderate the positive influence of contextual protective factors (Cleveland et al., 2010). Consequently, we explore the role of initial risk for substance use in affecting the youth program experience (i.e., implementation outcomes) and participant outcomes. In this study, we consider initial risk as those preexisting intervention recipient characteristics (early experimentation, single-parent household, SES) not changeable by the intervention and shown by prior research to relate to later substance use. Understanding these complex relationships has the potential to better inform program development, community policy, and implementation processes of evidence-based programs.

### The Importance of Implementation Quality for Participant Outcomes

Although the research literature and implementation practice describe many components and measures of implementation quality, we focus on understanding the impact of adherence, facilitation quality, and participant responsiveness. With the community coalition-driven decision-making and implementation process which promoted acceptability and appropriateness, and community buy-in, the three fidelity characteristics were deemed most likely to be important by project investigators and the characteristics on which data was collected.

#### Adherence

Researchers typically describe adherence as the degree to which planned curricular content was delivered (Dusenbury, 2003). It is logical to expect that replicating program outcomes is more likely when more of the planned program content is delivered, and in fact, many studies have demonstrated this. Positive associations between adherence and improved participant outcomes have been replicated in treatment settings (Collyer et al., 2020; Gifford et al., 2015; Hill & Owens, 2013; Hogue et al., 2008), school-based prevention (Cross et al., 2015; Dowling & Barry, 2020; Pettigrew et al., 2015; Shapiro et al., 2018), and family-focused prevention programs (Crowley et al., 2014; Hill & Owens, 2013; Véronneau et al., 2016). Yet some studies have shown no relationship or have suggested that the association may be more complicated. For example, several studies of evidence-based treatment and prevention programs have found no direct association between adherence and participant outcomes (Cantu et al., 2010; Hogue et al., 2008; Liber et al., 2010; Southam-Gerow et al., 2021). A variety of factors have been hypothesized as reasons for the lack of association, such as levels of participant engagement as measured by patient-therapist alliance ratings (Liber et al., 2010) and lack of variability in adherence. Our study adds value to this literature by further examining main and moderated effects of adherence, an important implementation outcome measure of fidelity, on participant outcomes; it is possible that formerly observed heterogeneity of program outcomes exists due to subgroup characteristics and how subgroup characteristics may interact with adherence.

#### Facilitation Quality

Facilitation quality, another measure of fidelity (Proctor et al., 2011), could be operationalized in many ways—often observers respond to a series of questions that describe qualities of how the facilitators or therapists implemented the program, such as their emotional responsiveness, pacing, and incorporating active learning strategies (Cross et al., 2015; Spoth et al., 2011a, b). At other times, facilitators self-report (Wray et al., 2018) or participants rate the quality of facilitation (Hostager et al., 2003). Although there are instances of facilitation quality not impacting youth outcomes, particularly in treatment settings (Hogue et al., 2008; Southam-Gerow et al., 2021), much of the research points to positive associations between facilitation quality and improved participant outcomes in school-based (Cross et al., 2015) and community-based prevention programs (Barbee et al., 2021) and also treatment-oriented settings (Collyer et al., 2020; Gifford et al., 2015). Our study adds to this literature by examining the role of facilitation quality, a type of implementation outcome, in predicting later participant outcomes, and further investigates the role of youth risk and facilitation quality to understand possible subgroup differences in program effects. Heterogeneity of effects due to subgroup differences could explain some of the mixed findings in the literature regarding facilitation quality and participant outcomes (Damschroder et al., 2022; Glasgow, et al., 2019a, b). For example, the quality of the facilitator may not matter for lower-risk youth; however, facilitation quality may strongly and positively associate with youth participant outcomes for high-risk youth (Miranda & Bostrom, 1999).

#### Participant Responsiveness

Researchers also describe participant responsiveness as another important fidelity characteristic (Proctor et al., 2011). Fewer studies focus on this implementation factor, yet existing empirical research suggests its importance for participant outcomes. For instance, one study of a school-based pregnancy prevention curriculum found that two measures of participant responsiveness—group cohesion and facilitator alliance—were associated with improvements participant attitudes and knowledge (Barbee et al., 2021). In a different study of a school-based obesity prevention program, participant responsiveness was one implementation outcome in a combined measure that positively associated with intentions to eat healthily, eat less junk food, and exercise more (Little et al., 2015). Additionally, qualitative research with adolescents has highlighted the importance of participant responsiveness for positive participant outcomes (Tirrell et al., 2021). Consequently, this study also examines the role of participant responsiveness in predicting participant substance use (Proctor et al., 2011), as well as how participant responsiveness may be linked differentially to outcomes (Damschroder et al., 2022; Glasgow et al., 2019a, b) across participant subgroups based on risk. It is possible that participant responsiveness may differentially predict youth outcomes for low-versus high-risk youth.

### Current Study

This current study moves the field of implementation and dissemination science further by testing a universal, voluntary, family-focused prevention program for differential outcomes for subgroups of intervention recipients. This current study draws from the PROSPER Partnership Model (Spoth et al., 2004) to better understand the potential for differential effects in universal prevention programs (Damschroder et al., 2022; Glasgow et al., 2019a, b; Offord, 2000) due to the complex association among levels of initial youth risk and the occurrence of implementation outcomes with program outcomes.

## Methods

### PROSPER Study Design

Participants in the study attended the family-focused component of the broader PROSPER trial that also included school-based prevention programming (*N* = 10,849). The PROSPER trial was a community-based, randomized study designed to test a novel system for delivering substance use prevention programming to middle school students in non-metropolitan areas of Iowa and Pennsylvania (Spoth et al., 2004). The PROSPER site selection criteria included (a) total school district enrollment between 1301 and 5200 students located in non-metropolitan areas, (b) at least 15% of students eligible for free or reduced-price lunches, (c) fewer than 50% of residents employed by or attending a university, (d) no involvement in other university-based prevention research projects, and (e) willingness of both the Extension Educator and School District to participate, including randomization to the intervention or control condition (Spoth et al., 2004).

This partnership-based prevention system involved three integrated tiers that supported evidence-based program selection, implementation, evaluation, and sustainability with the goal of preventing substance misuse and reducing risk for other problems. Tier one included a community-based team of local stakeholders; tier two included Prevention Coordinators from the Cooperative Extension System that linked community teams, with tier three involving university researchers, along with state-level coordinators and administrators. The original trial involved 28 school districts within the two states, Pennsylvania and Iowa, with half randomized to participate in this partnership-supported prevention system and the other half operated as a control group. Communities were block-randomized based on size and geographic location.

Sequential cohorts of sixth graders were involved in receiving intervention programming starting in 2002 with the first two cohorts providing data to assess impact from the PROSPER system. This paper focuses on the participants of the family-focused program, which comes from the communities randomized to receive the PROSPER intervention system.

### PROSPER Intervention Procedures

For school districts assigned to the intervention condition, there were two programs from a menu they could select: (1) a family-focused intervention targeting sixth-grade students and their caregivers, and (2) a school-based curriculum delivered to seventh-grade students. All intervention communities selected the Strengthening Families Program: 10–14 (SFP: 10–14) as the family-focused component, an intervention designed to enhance family functioning and youth resilience. Participation in the family program was voluntary, with 17% of eligible families choosing to enroll. The following year, all seventh-grade students in intervention communities received evidence-based preventive interventions (EBIs) during regular school hours.

The SFP 10–14 program aims to reduce adolescent substance use and other problem behaviors by improving parent–child relationships, enhancing parenting skills, and fostering prosocial behaviors and peer resistance skills in youth (Crowley et al., 2014; Demarsh & Kumpfer, 2013; Spoth et al., 2011a, b). The program consisted of seven weekly family sessions conducted during the sixth grade. Each session was led by three trained facilitators: one working with the parents, two working with the youth during their separate sessions, and all three leading the combined parent-youth sessions. Previous studies found that the specific impact of SFP 10–14, alongside the school-based program, led to a 5% decrease in underage drinking among participants (Crowley et al., 2014).

### PROSPER Research Procedures

#### Implementation Outcomes

Implementation outcomes of SFP:10–14 were assessed by trained observers (Spoth et al., 2007, 2011a, b), with youth, parent, and family sessions observed separately. Observation forms were divided into three sections: adherence, facilitation quality, and participant responsiveness. Approximately 25% of sessions had reports from two independent observers to ensure inter-rater reliability; all three measures showed an average correlation above 0.80 between observers (Spoth et al., 2011a, b). Adherence, facilitation quality, and participant responsiveness scores were averaged across all sessions at the community and cohort levels for analyses.

#### Student Surveys

Students’ substance use outcomes, initial risk status at baseline, and other individual characteristics were collected through an annual survey of students using machine-scored, written questionnaires administered in schools (Spoth et al., 2011a, b). The baseline survey occurred in the fall semester of sixth grade for each cohort (in 2002 for Cohort 1 and in 2003 for Cohort 2). Follow-up surveys for both cohorts were administered every spring semester through 12th grade. For this paper, we focus on early substance use as reported in early high school (ninth grade, in 2006–2007), as it represents the most immediate assessment of intervention impact following the completion of programming.

### Measures

#### Measures of Implementation Outcomes

Three measures were used to document the implementation outcomes of family-focused program implementation. *Adherence* measured the extent to which facilitators followed the core components of the intervention. For the SFP 10–14 family intervention, adherence was assessed by trained observers who independently rated sessions using checklists to document whether prescribed content and activities were delivered. Observers marked “Yes” or “No” to indicate whether specific content items were covered verbally or if a particular activity was conducted as outlined in the program protocol. Adherence scores were calculated as the proportion of prescribed content completed (Spoth et al., 2007).

*Facilitation quality* measured the facilitators’ positive behaviors like friendliness and responsiveness to participants’ questions. Sample items from the observation forms included questions such as, “How accepting and friendly was the group leader?” and “To what extent was the group leader unable to deal effectively with questions?” Observers rated responses on a scale from 0 to 4, with 0 indicating “Unfriendly” or “Never,” and 4 indicating “Friendly” or “Frequently.” Negatively worded items were reverse-coded so that higher scores reflected more positive facilitation quality. Final facilitation scores were calculated by averaging relevant items for each facilitator during a session.

*Participant responsiveness* was measured by observer ratings of participation. The participation measure represents the level of engagement and involvement of children and families in the sessions (Spoth et al., 2011a, b). Participation was rated by observers using two items: the level of active participation in session activities and the degree of interest displayed by participants. Both items were rated on a Likert scale from 0 to 4, where 0 indicated “Little participation/Not interested,” and 4 indicated “Active participation/Very interested.” The final participant responsiveness score for each session was calculated by averaging these two items.

#### Outcomes: Substance Use in 9th grade

Outcomes assessed when participants were in the ninth grade included measures of gateway and illicit substance use, as well as lifetime and past-month use of various substances. The Gateway Substance Initiation Index (SII-G) was calculated by summing lifetime use of three substances: (1) alcohol, (2) cigarettes, and (3) marijuana (or hashish). The Illicit Substance Initiation Index (SII-I) was calculated by summing lifetime use of five substances: (1) methamphetamine, (2) ecstasy, (3) marijuana or hash,^1^ (4) drugs or prescribed medications, and (5) Vicodin, Percocet, or OxyContin. New-user measures were defined for participants who reported no lifetime substance use at baseline but indicated substance use by ninth grade. Three new-user measures were included for analysis: drunkenness, cigarette use, and marijuana use. Additionally, “current” substance use was measured through past-month use of alcohol and cigarettes, which have relatively higher rates of use in ninth grade (Spoth et al., 2011a, b).

#### Initial Risk Status of Youth

Risk status was determined using five binary factors reported by participants at baseline: lifetime use of gateway substances (alcohol, cigarettes, or marijuana), engagement in problem behaviors (at least 2 out of 12 possible behaviors in the past year), eligibility for the free or reduced-cost school lunch program, lower family cohesion (dichotomized), and living with one or no biological parents (compared to living with both biological parents). Students were considered at higher risk if they reported either (a) three or more of the five risk factors, or (b) two risk factors, if one of the two was either gateway substance use or conduct problems (Spoth et al., 2017).

#### Other Control variables

Age and gender of the youth participants were included as demographic control variables given relevance to early substance use. The number of sessions attended was also included as a covariate. Additionally, the proportion of high school graduates reported by the Census (U.S. Census Bureau, 2000) within the community at baseline was included to represent general contextual risk.

### Data Analysis Plan

The final dataset for this analysis comprises substance use outcomes and individual characteristics, as well as cohort- and community-level measures of family-focused program implementation quality and community-level covariates. The sample (*N* = 673) includes participants who attended at least one of the seven sessions of the family-focused SFP program within the intervention community and completed both the baseline and follow-up surveys with substance use outcomes. Approximately 13% of baseline respondents were missing follow-up data on substance use outcomes.

Following descriptive analyses, we estimated unconditional random-intercept-only multilevel models (null models) to calculate intra-class correlations (ICCs) for the substance use outcomes. The ICC for new cigarette use was slightly above 0.05, while the others ranged from 0.01 to 0.03. Despite the relatively low ICCs, we proceeded with multilevel modeling models with random intercepts to account for the nested data structure (individuals within communities) and ensure consistency across outcomes. To account for potential non-independence of observations within clusters, standard errors were clustered at the community-cohort level.

Initial analysis examined the overall linkage between family-focused program implementation quality in cohort-community level (Level 2) and youth’s initial risk status (Level 1) and the individual-level substance use outcomes (Level 1). The three implementation quality measures were standardized in the model, with each measure tested in separate models to assess its unique effect and to compare the effects across sub-measures. For testing the moderation role of implementation quality, the interaction between implementation quality and youth’s initial risk status was included as the primary predictor. This interaction term enabled tests for whether the impact of implementation quality on substance-use outcomes varied based on the youth’s initial risk status, highlighting any differential effects of the level of program implementation depending on risk level.

## Results

Summary statistics for the individual-level outcome and controls, implementation quality variables at the community and cohort levels, and community-level control variables are presented in Table 1. For outcome variables, the Gateway Substance Initiation Index ranged from 0 to 3 (*M* = 1.33, SD = 0.97), and the Illicit Substance Initiation Index ranged from 0 to 5 (*M* = 0.61, SD = 1.13). Among new users (excluding baseline users), 32% reported first-time drunkenness, 30% reported new cigarette use, and 17% initiated marijuana use. In terms of past-month use, 36% reported alcohol use and 17% reported cigarette use.

**Table 1:** Summary statistics

	*N*	Mean	SD	Min	Max
A. Outcome variables					
*Substance use index*					
Gateway Substance Initiation Index (SII-G)	673	1.33	0.97	0	3
Illicit Substance Initiation Index (SII-I)	667	0.61	1.13	0	5
*New user*					
Drunkenness	608	0.32	0.47	0	1
Cigarettes	581	0.3	0.46	0	1
Marijuana	610	0.17	0.38	0	1
*Past month use*					
Alcohol	672	0.36	0.48	0	1
Cigarette	672	0.17	0.37	0	1
B. Control variables					
*Individual level*					
Gender	673	0.5	0.5	0	1
Age	673	15.21	0.36	14.4	16.5
Risk status (1 = with risk, 0 = no risk)	673	0.24	0.43	0	1
Number of sessions attended	673	5.41	1.87	1	7
*Community level*					
% of high school graduates	14	40.39	5.40	33.35	50.85
C. Implementation quality (cohort-community level)					
Adherence	27	0.91	0.04	0.84	0.97
Facilitation quality	27	3.69	0.16	3.31	3.94
Participant responsiveness	27	3.58	0.19	3.06	3.93

The individual-level control variables show that gender is evenly distributed, and the average age of participants is 15.21 years (SD = 0.36). Participants attended an average of 5.41 sessions (SD = 1.87). At baseline, 24% were classified as high-risk. At the community level, the percentage of high school graduates averaged 40.39% (SD = 5.40), ranging from 33.35% to 50.85%. Implementation quality indicators across 27 communities and cohort^2^ were as follows: adherence averaged 0.91 (SD = 0.04; range = 0.84–0.97), facilitation quality averaged 3.69 (SD = 0.16; range = 3.31–3.94), and participant responsiveness averaged 3.58 (SD = 0.19; range = 3.06–3.93).

Results from regression models testing the effect of implementation quality on substance use outcomes are provided in Tables 2 and 3 broken down by study outcomes and implementation quality predictor. As shown in Table 2, initial risk level was significantly linked to both substance initiation indices. However, implementation quality of the family-focused program, measured through adherence, facilitation quality, and participant responsiveness, was not significantly associated with substance initiation indices (in terms of main effects) (Table 2). For new user and past month substance use outcomes (Table 3), facilitation quality was positive but marginally (*p* < 0.1) associated with reductions in new instances of drunkenness, cigarette use, and past-month alcohol and use. Adherence scores and participant responsiveness showed no significant association with any of the substance use outcomes.

**Table 2:** Effect of implementation quality on Substance Initiation index

Variables	Gateway Substance Initiation index			Illicit Substance Initiation Index		
	(1)	(2)	(3)	(4)	(5)	(6)
Gender	− 0.07	− 0.07	− 0.07	− 0.03	− 0.03	− 0.03
	(0.07)	(0.07)	(0.07)	(0.06)	(0.07)	(0.06)
Age	0.28**	0.28**	0.28**	0.26**	0.26**	0.27**
	(0.11)	(0.11)	(0.11)	(0.11)	(0.11)	(0.11)
Risk status	0.76***	0.76***	0.76***	0.81***	0.81***	0.80***
	(0.07)	(0.08)	(0.08)	(0.12)	(0.12)	(0.12)
# Session attended	− 0.05***	− 0.05***	− 0.05***	0.00	− 0.00	0.00
	(0.02)	(0.02)	(0.02)	(0.02)	(0.02)	(0.02)
Adherence	− 0.01			− 0.01		
	(0.01)			(0.01)		
Facilitation quality		− 0.21			0.24	
		(0.22)			(0.20)	
Participant responsiveness			− 0.10			0.05
			(0.18)			(0.22)
% of HSG	0.02***	0.02**	0.01**	0.01	0.01	0.01
	(0.01)	(0.01)	(0.01)	(0.01)	(0.01)	(0.01)
Observations	663	663	663	667	667	667

Standard errors are clustered at the community-cohort level and reported in parentheses; *HSG* high school graduates; The Gateway Substance Initiation Index, Illicit Substance Initiation Index, and the three measures of intervention quality (adherence, facilitation quality, and participant responsiveness) are standardized

^*^p < 0.1, **p < 0.05, ***p < 0.001

**Table 3:** Effect of implementation quality on new substance use and past-month substance use (odds ratios)

Variables	(1)	(2)	(3)	(4)	(5)
	New drunkenness	New cigarette user	New marijuana user	Past month alcohol use	Past month cigarette use
Adherence	0.99	0.96	0.94	0.96	0.99
	(0.04)	(0.04)	(0.04)	(0.05)	(0.02)
Facilitation quality	0.34*	0.25*	0.48	0.15**	0.48
	(0.20)	(0.19)	(0.50)	(0.14)	(0.22)
Participant responsiveness	0.49	0.45	0.54	0.33	0.67
	(0.25)	(0.28)	(0.43)	(0.23)	(0.25)

Standard errors are clustered at the community-cohort level and reported in parentheses; each cell in this table represents odds ratios from separate specifications. Three measures of intervention quality (adherence, facilitation quality, and participant responsiveness) are standardized. The table reports only the effects of the three measures of intervention quality on substance use outcomes. Full results, including all covariates, are provided in the supplementary materials

^*^p < 0.1, **p < 0.05, ***p < 0.001

**Fig. 1:**
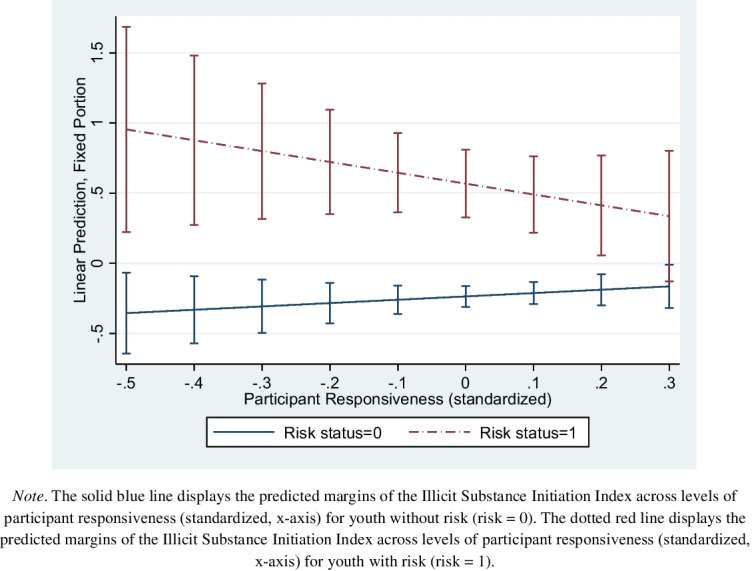
Predictive Margins of Illicit Substance Initiation Index with 95% confidence intervals

Regression models incorporating interaction terms revealed that implementation quality, as indicated by measures of participant responsiveness and facilitation quality, significantly moderates the relationship between initial risk status and youth substance use outcomes (Tables 4 and Table 5). Youth identified as being at high initial risk were shown to have elevated the Gateway Substance Initiation Index (SII-G) and the Illicit Substance Initiation Index (SII-I) in ninth grade (by 0.74 SD and 0.79 SD, respectively), compared to their peers without risk (Column 1 and 2 in Table 4). However, a significant interaction between participant responsiveness and risk factors was linked to a reduction in the Gateway Substance Initiation Index at ninth grade (by 0. 80 SD per 1 SD increase in participant responsiveness score) and the Illicit Substance Initiation Index (by 1. 06 SD) for high-risk youth. Figure 1 presents the marginal predictions of the Illicit Substance Initiation Index (models from columns 2 of Table 4) for youth with and without initial risk separately. The figure shows divergent patterns in the predicted Illicit Substance Initiation Index by youth risk status. As participant responsiveness increases, the predicted index decreases significantly for youth with high levels of initial risk (risk status = 1), whereas it remains relatively stable for youth not at risk (risk status = 0). This pattern suggests that higher-quality participant responsiveness is particularly effective in reducing illicit substance initiation among high-risk youth.

**Table 4:** Effect of participant responsiveness on substance use of children with initial high-risk levels

Variables	(1)	(2)	(3)	(4)	(5)
	SII-G	SII-I	New cigarette use	New marijuana use	Past month alcohol use
Risk status	0.74***	0.79***	2.72***	4.12***	3.14***
	(0.07)	(0.13)	(0.64)	(0.79)	(0.43)
Participant responsiveness	0.06	0.21	0.84	1.03	1.09
	(0.15)	(0.23)	(0.51)	(0.84)	(0.32)
Risk status* participant responsiveness	− 0.80**	− 1.06*	0.07**	0.17**	0.13**
	(0.39)	(0.51)	(0.09)	(0.12)	(0.13)
Observations	663	667	578	606	674

Standard errors are clustered at the community-cohort level and reported in parentheses. Participant Responsiveness, the Gateway Substance Initiation Index (SII-G) (Column 1), and the Illicit Substance Initiation Index (SII-I) (Column 2) are standardized. Columns 3–6 present odds ratios, as the substance use outcomes in these columns are binary

^*^p < 0.1, **p < 0.05, ***p < 0.001

**Table 5:** Effect of facilitation quality on Substance use of children with initial high-risk levels (odds ratios)

Variables	(1)	(2)
	New marijuana	Past month alcohol use
Risk status	4.03***	2.98***
	(0.77)	(0.77)
Facilitation quality	1.10	0.55
	(1.08)	(0.22)
Risk status*facilitation quality	0.11*	0.08**
	(0.12)	(0.07)
Observations	606	674

Standard errors are clustered at the community-cohort level and reported in parentheses; facilitation quality is standardized. This table presents odds ratios, as both the new marijuana use and past-month alcohol use outcomes are binary variables. This table presents only the specifications for outcomes with statistically significant effects. Full results for all outcomes are available upon request

^*^p < 0.1, **p < 0.05, ***p < 0.001

**Fig. 2:**
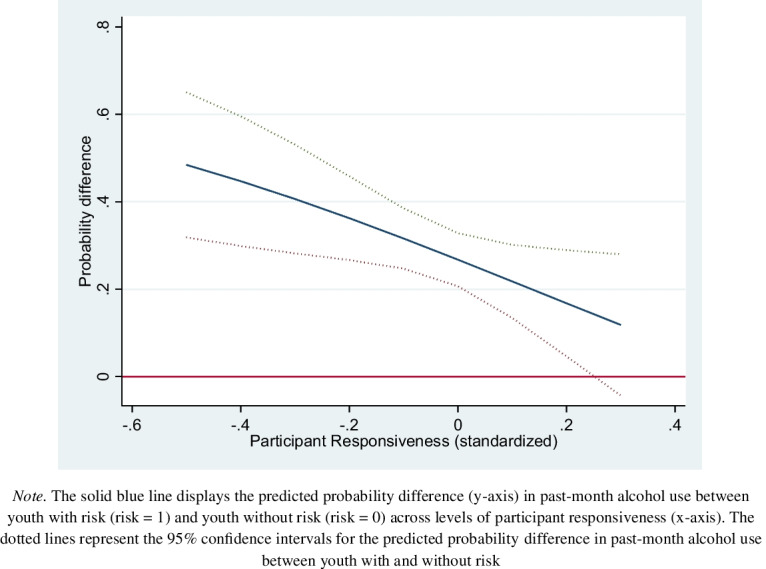
Probability difference in past-month alcohol use: youth with initial risk vs. no initial risk

Participant responsiveness also had differential associations with cigarette, marijuana, and past-month alcohol use in ninth grade depending on participants’ initial risk status (Table 4, Column 3–5). Results showed a 1 SD increase in participant responsiveness corresponds to lower odds of new cigarette-use (odd ratio = 0.07, roughly by 93%) and new marijuana use (odd ratio = 0.17) among ninth-grade students with initial risk status. Similarly, participant responsiveness works as a moderator lowering the probability of alcohol use and cigarette use for youth with initial high levels of risk. The graph in Fig. 2 illustrates the probability difference in past-month cigarette-use between youth with and without risk. The results indicate that the gap in probability of using cigarettes between youth with and without risk decreases as the participant responsiveness score in the family-focused program increases.

Facilitation quality was also found to be differentially linked with substance use outcomes based on initial risk, for marijuana use and past-month alcohol use in ninth grade (Table 5). As shown in Column 1 in Table 5, an increase in the facilitation quality of the family-focused program is associated with lower possibility of new marijuana use (odd ratio = 0.11) and past-month alcohol use (odd ratio = 0.08) in ninth grade for youth with risk. Notably, the results indicate that there were no significant linkages found between program adherence and the substance use outcomes in this study, either overall or differentially based on initial risk level.

Overall, the results suggest that implementation quality had no or only marginal direct effects on substance use outcomes on its own but served as a significant moderator between risk status and substance use outcomes. Among youth with high levels of initial risk, higher participant responsiveness was linked to reduced substance use for five of seven related outcomes, including gateway and illicit substance use, cigarette, marijuana initiation, and past-month alcohol use. Facilitation quality also was linked to lower new marijuana use and alcohol use for youth with high levels of initial risk. In contrast, implementation quality measures had lower or no association with substance use outcomes for youth not identified as higher risk.

## Discussion

Our findings contribute to the growing literature on implementation quality in prevention programming and youth development, specifically regarding substance use outcomes. In particular, these findings highlight the moderating role of fidelity characteristics, specifically facilitation quality and participant responsiveness, in the association between youth’s initial risk status and subsequent substance use, an especially important consideration when policy typically aims to prevent problems in those who face greater risks for those problems.

Consistent with previous studies (e.g. Cantu et al. (2010), Liber et al. (2010), Southam-Gerow et al. (2021)), our results found that the program adherence, which was uniformly high across communities (mean = 0.91, SD = 0.04), showed no significant association with outcomes, possibly due to limited variability in a more controlled intervention trial (Liber et al., 2010). Future studies with data that comprise greater variation in adherence quality, perhaps reflecting more realistic situations in services delivery, could further examine the potential differential link based on initial risk between adherence and substance use outcomes.

At the same time, the lack of findings related to adherence may indicate that more interactive and relational aspects of delivery, such as facilitation quality and participant responsiveness, are potentially more influential for high-risk youth. This aligns with recent implementation science that highlights the importance of relational and participatory elements in driving program effectiveness (Barbee et al., 2021; Cross et al., 2015; Gifford et al., 2015; Little et al., 2015). This suggests that engaging delivery methods may be particularly essential for reaching youth who often exhibit lower responsiveness to interventions (Montero-Marin et al., 2023) and are particularly sensitive to environmental influences (Luthar et al., 2014; Thibodeau et al., 2016).

Relatedly, attendance at SFP: 10–14 may be an important mediator linking implementation quality to youth substance use outcomes. Prior PROSPER research showed that higher attendance improves family functioning and youth protective factors, thereby reducing substance use initiation (LoBraico et al., 2021), a pattern consistent with our finding that greater session attendance was associated with lower substance use. Attendance may both influence and be influenced by facilitation quality and participant responsiveness, and its effects may vary by youth risk status as higher-risk families often face greater participation barriers but may benefit most from high-quality, higher-dosage interventions (Arthur et al., 2007; Brody et al., 2006).

Several mechanisms may underlie the moderating effect of participant responsiveness in the link between risk status and substance use outcomes (Glasgow et al., 2019a, b). A meta-analysis by Allen et al. (2003) summarizes evidence demonstrating that both peer and family influences are central predictors shaping adolescents’ substance use decisions. Especially for high-risk youth, who often experience peer rejection and limited social resources, sensitivity to peer influence may be heightened during adolescence (Dishion et al., 2014; Dodge et al., 2006; Van Ryzin & Dishion, 2013). In this context, integrating youth into supportive peer groups characterized by high participant responsiveness through family-focused interventions may be particularly protective by simultaneously reshaping peer exposure (Barbee et al., 2021; Brown et al., 2009; Ennett et al., 1994). Actively participating in family-focused programs alongside lower-risk peers may encourage prosocial peer bonding, which in turn can reduce engagement in antisocial behaviors and substance use (Ennett et al., 2006; Kellam et al., 2008; Van Ryzin et al., 2023).

The finding that high-risk youth benefited more from high-quality facilitation than their lower-risk counterparts could suggest that skilled facilitators, through behaviors marked by responsiveness, warmth, and clarity, can create inclusive environments that amplify the intervention’s impact (Brown et al., 2009). Relational competence of facilitators may help fulfill these youths’ psychological needs for acceptance and safety (Sanders et al., 2016), and positive relationships with facilitators can enhance the resilience process among youth with high levels of initial risk, improving outcomes and ultimately increasing the intervention’s overall impact (Liebenberg, 2020; Miranda & Bostrom, 1999). Our findings suggest that by cultivating trust and modeling empathic responsiveness, facilitators may enable high-risk youth to reinterpret supportive interventions as credible and affirming rather than judgmental or imposed (Braciszewski et al., 2018; Durlak et al., 2011; Miller & Rollnick, 2013). This relational reframing may especially be critical in the context of substance use prevention for high-risk youth (Damschroder et al., 2022; Glasgow et al., 2019a, b; Hawkins et al., 2010).

More generally, the family-focused components of the intervention may represent an additional mechanism benefiting high-risk youth from families with lower cohesion or lower socioeconomic status (Arthur et al., 2007; Brody et al., 2006). By increasing parental resources, parenting skills, and awareness around early substance use—alongside interaction with other parents from lower-risk families and high-quality facilitators—the intervention may enhance parents’ capacities to guide adolescents’ decision-making (Allen et al., 2003; Kumpfer & Alvarado, 2003; Sandler et al., 2011). Greater parental awareness and involvement, in turn, may positively influence adolescents’ substance use choices, consistent with prior evidence on the protective role of family influence (Allen et al., 2003).

It is important to note that these findings exist within a prevention delivery system that involved a partnership with a research institution and state cooperative extension. This could have helped provide both better support for quality implementation as well as greater monitoring than would otherwise typically occur in implementations by free-standing, independent, and often isolated community organization settings. While it may be harder to monitor and control in other settings, these findings provide further evidence that implementation quality tracking is important to make common practice. Higher-risk students will need quality prevention services, and any investment in assuring higher quality of family programming would likely pay off in the long run given the problems and costs associated with untreated substance use in late adolescence/early adulthood (Arria et al., 2018; Erskine et al., 2015; Rockett et al., 2005). There is potential for prevention systems to provide a local, supportive accountability mechanism which facilitates sustained higher quality implementation and outreach, so projects such as PROSPER could be better situated to provide the infrastructure that can best serve those most in need of preventive services.

It is also worth noting that the program featured here—SFP: 10–14—entails universal prevention albeit voluntary for those whose families agree to participate. Universal prevention can enable delivery of services to those at higher risk for substance misuse without the potential stigma that could occur through indicated or targeted preventive programming. Universal programming can also enable reaching those at risk for antisocial behavior who may otherwise not have been identified (Greenberg & Abenavoli, 2017). Compared to selective or targeted programs, universal programming also brings together participants across different risk levels, thereby allowing higher-risk students to benefit from positive peer influences and perceived acceptance from lower-risk peers (Spoth et al., 2006). This study represents services that occurred through natural processes, involving adolescents and their families who were available and open to receiving the program. Within that pool of participants, these findings provide additional information on key linkages between differential risk and program implementation characteristics potentially influencing early substance use.

The findings of this study should be interpreted considering several limitations. First, the sample predominantly comprised white, non-metropolitan participants, which constrains the generalizability of the results; associations may look different in more diverse or urban populations across different national contexts. Second, all participants in the SFP: 10–14 group also previously participated in a school-based EBI. Though prior research identified the unique impact of the SFP: 10–14 program on youth outcomes (Crowley et al., 2014), it is possible that effects would look different without the school-based program implementation. Future research should examine the quality of implementation of the school-based component and its interaction with the family-based intervention to more comprehensively understand the mechanisms driving observed substance use outcomes. Finally, the data used in this study were collected in 2006–2007, and thus, the findings should be interpreted within the context of that period. Given ongoing and rapid changes in substance use patterns, policy, and youths’ social contexts (including the rise of social media), future research using more recent data will be important to assess the extent to which these patterns hold in contemporary settings.

In conclusion, these findings have important implications for intervention design and delivery. Emphasizing participatory and interactive approaches, and training facilitators to create inclusive and affirming environments, can enhance program impact, particularly for high-risk youth. Rather than conceptualizing implementation quality solely in terms of protocol adherence, it is crucial for practitioners to prioritize the relational quality of program delivery and to promote inclusion as core components of effective practice. In the field, improving facilitation quality and participant responsiveness requires a data-driven approach, including systematic monitoring of implementation quality and participation, integration of community feedback into ongoing evaluation, and evidence-based facilitator training. These efforts can foster more inclusive environments that support sustained engagement among youth with high levels of initial risk and their families.

## Supplementary information

Below is the link to the electronic supplementary material.

ESM 1 (DOCX 34.4 KB)

## Footnotes

1 Marijuana was included in both the Gateway Substance Initiation Index (SII-G) and the Illicit Substance Initiation Index (SII-I) to maintain consistency with the original PROSPER measurement protocol. Theoretically, marijuana fits both classifications, as it is commonly viewed as a gateway substance and is also classified as an illicit drug (Shukla, 2013) at the time of the data collection.

2 Of the 28 total intervention communities (comprising 14 communities with two cohorts each), one cohort in a community lacked implementation quality data and was therefore excluded from the analysis.
