# Supplementary material for: The Role of Program Implementation Quality in Family-Focused Substance-Use Prevention for Youth with Multiple Risks: PROSPER Project
Source: Prev Sci. 2026 May 23;27(6):876–89. doi: 10.1007/s11121-026-01929-9 (PMC13433459; doi:10.1007/s11121-026-01929-9)
Supplement: Supplementary file 1 [file 11121_2026_1929_MOESM1_ESM.docx]

Table S1. Main effect on new substance use (Odd ratio)

|  | New Drunk | | | New Cigarette user | | | New Marijuana user | | |
| --- | --- | --- | --- | --- | --- | --- | --- | --- | --- |
|  | (1) | (2) | (3) | (4) | (5) | (6) | (7) | (8) | (9) |
|  |  |  |  |  |  |  |  |  |  |
| Gender | 0.73 | 0.74 | 0.74 | 0.66* | 0.67* | 0.67* | 1.18 | 1.21 | 1.21 |
|  | (0.15) | (0.15) | (0.15) | (0.15) | (0.15) | (0.15) | (0.28) | (0.28) | (0.29) |
| Age | 1.69** | 1.69** | 1.70** | 2.45*** | 2.46*** | 2.46*** | 1.82** | 1.81** | 1.81** |
|  | (0.36) | (0.37) | (0.37) | (0.68) | (0.68) | (0.68) | (0.51) | (0.51) | (0.51) |
| Risk status | 3.39*** | 3.33*** | 3.36*** | 2.92*** | 2.85*** | 2.88*** | 4.33*** | 4.24*** | 4.26*** |
|  | (0.51) | (0.51) | (0.51) | (0.70) | (0.69) | (0.70) | (0.87) | (0.83) | (0.85) |
| # Session attended | 0.93* | 0.93* | 0.93* | 0.85** | 0.85** | 0.85** | 0.98 | 0.98 | 0.98 |
|  | (0.04) | (0.04) | (0.04) | (0.06) | (0.06) | (0.06) | (0.05) | (0.06) | (0.06) |
| Adherence | 0.99 |  |  | 0.96 |  |  | 0.94 |  |  |
|  | (0.04) |  |  | (0.04) |  |  | (0.04) |  |  |
| Facilitation Quality |  | 0.34* |  |  | 0.25* |  |  | 0.48 |  |
|  |  | (0.20) |  |  | (0.19) |  |  | (0.50) |  |
| Group participation |  |  | 0.49 |  |  | 0.45 |  |  | 0.54 |
|  |  |  | (0.25) |  |  | (0.28) |  |  | (0.43) |
| % of HSG | 1.01 | 1.01 | 1.01 | 1.01 | 1.01 | 1.01 | 1.04 | 1.03 | 1.03 |
|  | (0.02) | (0.02) | (0.02) | (0.03) | (0.02) | (0.02) | (0.02) | (0.02) | (0.02) |
|  |  |  |  |  |  |  |  |  |  |
| Observations | 606 | 606 | 606 | 578 | 578 | 578 | 606 | 606 | 606 |
| Number of groups | 27 | 27 | 27 | 27 | 27 | 27 | 27 | 27 | 27 |

*Note*. Standard errors are clustered at the community-cohort level and reported in parentheses.

* p < 0.1, ** p < 0.05, *** p < 0.001.

Table S2. Main effect on Past month’s substance use (Odd ratios)

|  | Past Month Alcohol Use | | | Past Month Cigarette Use | | |
| --- | --- | --- | --- | --- | --- | --- |
|  | (1) | (2) | (3) | (4) | (5) | (6) |
|  |  |  |  |  |  |  |
| Gender | 0.75 | 0.76 | 0.76 | 0.69** | 0.70** | 0.69** |
|  | (0.21) | (0.21) | (0.21) | (0.12) | (0.12) | (0.12) |
| Age | 1.71** | 1.72** | 1.71** | 1.11 | 1.11 | 1.11 |
|  | (0.45) | (0.46) | (0.45) | (0.24) | (0.24) | (0.24) |
| Risk status | 3.99*** | 3.91*** | 3.96*** | 3.26*** | 3.24*** | 3.25*** |
|  | (0.79) | (0.76) | (0.79) | (0.43) | (0.44) | (0.44) |
| # Session attended | 0.94 | 0.94 | 0.94 | 1.02 | 1.02 | 1.02 |
|  | (0.06) | (0.06) | (0.07) | (0.05) | (0.05) | (0.05) |
| Adherence | 0.96 |  |  | 1.00 |  |  |
|  | (0.05) |  |  | (0.03) |  |  |
| Facilitation Quality |  | 0.15** |  |  | 0.48 |  |
|  |  | (0.14) |  |  | (0.22) |  |
| Group participation |  |  | 0.33 |  |  | 0.67 |
|  |  |  | (0.23) |  |  | (0.25) |
| % of HSG | 1.05* | 1.04* | 1.04* | 1.04*** | 1.04*** | 1.04** |
|  | (0.02) | (0.02) | (0.02) | (0.01) | (0.01) | (0.02) |
|  |  |  |  |  |  |  |
| Observations | 673 | 673 | 673 | 674 | 674 | 674 |
| Number of groups | 27 | 27 | 27 | 27 | 27 | 27 |

*Note.* Standard errors are clustered at the community-cohort level and reported in parentheses.

* p < 0.1, ** p < 0.05, *** p < 0.001.
